# Training and match demands differ between the regular season and finals in semi-professional basketball

**DOI:** 10.3389/fspor.2022.970455

**Published:** 2022-08-25

**Authors:** Jodie A. Palmer, Rodrigo Bini, Daniel Wundersitz, Michael Kingsley

**Affiliations:** ^1^Holsworth Research Initiative, La Trobe Rural Health School, La Trobe University, Bendigo, VIC, Australia; ^2^Department of Exercise Sciences, Faculty of Science, University of Auckland, Auckland, New Zealand

**Keywords:** accelerometry, high-intensity intermittent exercise, team sports, athletic performance, AvF_NET_

## Abstract

Basketball competitions often include a scheduled regular season followed by knock-out finals. Understanding training and match demands through the season can help optimize performance and reduce injury risk. This study investigated whether training and/or match demands differed between the regular season and finals, and whether these differences were dependent on player role. Average session intensity and volume and durations of relative exercise intensities (inactive, light, moderate-vigorous, maximal, supramaximal) were quantified during training sessions and matches using accelerometry in two semi-professional basketball teams (*n* = 23; 10 women, 13 men). Training and match demands were compared between the regular season (training: 445 observations; matches: 387 observations) and finals (training: 113 observations, matches: 75 observations) with consideration of player role (starters, in-rotation bench, out-rotation bench). During finals matches, starters received 4.4 min more playing time (*p* = 0.03), performed 14% more absolute maximal activity (*p* < 0.01) and had 8% less relative inactive time (*p* = 0.02) when compared to the regular season. Out-rotation bench players received 2.1 min less playing time (*p* < 0.01), performed 33% less absolute maximal activity (*p* = 0.01) and 57% less absolute supramaximal activity (*p* < 0.01) in finals when compared to the regular season. During finals training sessions, average training intensity was 5% higher (*p* = 0.02), absolute moderate-vigorous activity was 3% higher (*p* = 0.04), relative maximal activity was 12% higher (*p* < 0.01), and relative inactive time was 5% lower (*p* = 0.03) when compared to the regular season. These findings suggest starters need to be physically prepared for greater match demands during finals, while out-rotation bench players should supplement their training during finals with extra supramaximal activity to maintain their conditioning levels for matches.

## Introduction

Many team sports, such as Australian football, netball, rugby league and soccer, are played in a seasonal format, consisting of a regular season where teams aim to finish with the highest ranking possible, and finals where the top-ranked teams play knock-out matches until one winning team remains. To maximize performance and reduce injury risk, it is important that players are physically prepared for the training and match demands they are likely to experience (Ziv and Lidor, 2009; Gabbett et al., 2016). Basketball training and match demands have been quantified using various methods, such as heart rate monitoring (Abdelkrim et al., 2010), video-based time-motion analysis (Scanlan et al., 2011), and more recently, accelerometry-based time-motion analysis (Staunton et al., 2018a,b). Accelerometry-based time-motion analysis has particular utility for quantifying basketball training and match demands due to its high sampling rate and ability to quantify activity in 3-dimensions (Staunton et al., 2018a). Basketball is characterized as a highly intermittent sport, with frequent, short periods of high-intensity anaerobic activity interspersed among recovery periods of lower-intensity activity (Staunton et al., 2018a; Petway et al., 2020; Palmer et al., 2021). Players have been reported to spend 63–93% of live time at heart rates above 85% of maximal heart rate, and cover 2–7 km per match with frequent accelerations, decelerations and changes of direction (Stojanović et al., 2018; Petway et al., 2020). Various factors, including the level of competition (more moderate-intensity match activity and greater training demands at higher levels of competition; Scanlan et al., 2011; Ferioli et al., 2020; Palmer et al., 2021), playing position (more supramaximal match activity performed by back-court players; Staunton et al., 2018a) and player role (greater match demands for starting players compared to in-rotation and out-rotation bench players; Palmer et al., 2021) can influence these training and match demands. It is also plausible that training and match demands differ as players move from the regular season into finals.

In comparison to regular season, heightened match importance and increased opposition quality during finals might lead to higher training and match demands. Finals matches have been shown to elicit greater demands than regular season matches in other team sports (Aughey, 2011; Mangan et al., 2019). For example in Australian football, finals matches elicited 11% greater total distance, 9% greater high-intensity running distance and 97% more maximal accelerations than regular season matches (Aughey, 2011). In Gaelic football, total distance and high-speed running distance were up to 44–63% greater during the All-Ireland Championships (premier playoff competition at the end of the season) than during the rest of the season (Mangan et al., 2019). However, no differences in session rating of perceived exertion (sRPE) during matches were found between the regular season and playoffs in professional basketball (Ferioli et al., 2021). In the aforementioned study, all players (*n* = 10) were combined into one group without consideration for player role due to sample size limitations. As match demands in basketball are dependent on player role (i.e., starter, in-rotation bench, out-rotation bench; Palmer et al., 2021), differences in match demands between the regular season and finals might be influenced by player role. Therefore, player role is likely to be an important consideration when evaluating the demands of basketball match-play between the regular season and finals. Additionally, sRPE might not be sensitive enough to detect changes between the regular season and finals. Objective variables quantifying on-court activity might be more sensitive to such changes; however, no studies have assessed this in basketball.

Therefore, the aims of this study were to determine: (1) if time-motion analysis-derived training and match demands differ between the regular season and finals in basketball, and (2) if these differences are dependent on player role. Based on previous findings, it was hypothesized that training and match demands for starters would be greater during finals compared to the regular season, while training and match demands for bench players would be less.

## Materials and methods

### Participants

A sample size calculation for linear mixed models was conducted using R (R Core Team, 2021, Vienna, Austria) using the sjstats package (RDocumentation, 2022). With 23 players, effect size of 0.40, power of 0.80 and significance level of 0.05, at least 14 observations per subject were required, resulting in a total sample size of 331. Differences in time-motion analysis-derived demands between the regular season and finals have not been assessed in basketball, so the anticipated effect size was determined from analyses of other sports. In Australian football, effect sizes for differences in high-intensity running distance and number of maximal accelerations between the regular season and finals were 0.29 and 1.30, respectively (Aughey, 2011). Subsequently, 0.40 was selected as a conservative estimate of anticipated effect size, which also reflects the center of the small effect size range of 0.20–0.60 (Hopkins, 2002). 23 basketball players were recruited from a women's and men's semi-professional team belonging to the same basketball organization (Table 1). Both teams competed in the Australian 2019 NBL1 season (second-highest competition level in Australia). All players provided written informed consent prior to participating. Ethical approval was granted by the La Trobe University Human Research Ethics Committee (HEC15-088) in accordance with the Declaration of Helsinki.

**Table 1:** Participant characteristics.

**Team**	**Players**	**Age (years)**	**Height (cm)**	**Mass (kg)**	**Regular season**		**Finals**	
					**Training sessions**	**Matches**	**Training sessions**	**Matches**
Women	10	28.5 ± 5.4	176 ± 10	76.5 ± 19.5	26	20	4	3
Men	13	26.8 ± 5.2	192 ± 8	96.2 ± 16.4	24	20	8	4

### Study design

This study was observational. During pre-season, players completed a modified Yo-Yo Intermittent Recovery 1 Test (IR1) while wearing a 100 Hz triaxial accelerometer (GT9X Link; Actigraph, FL). This process enabled the estimation of the average net resultant force (AvF_NET_) acting on their body in a range of walking and running speeds to generate individualized intensity distribution bands (Staunton et al., 2018a). Throughout the competitive season, players wore the same accelerometer during on-court team training sessions and matches. The accelerometer was positioned between the player's scapulae using a tightly-fitted sports vest as described previously (Wundersitz et al., 2015). Accelerometers have shown acceptable intra- and inter-device reliability in laboratory and team sport settings for quantifying movement demands (Kelly et al., 2015; Nicolella et al., 2018).

### Procedures

On-court training sessions were monitored throughout the competitive season, occurring 1–2 times a week on Tuesday and Thursday evenings for 90 (range 84–100) min per session. Training sessions typically consisted of shooting drills, ball-handling drills, dynamic game-based drills, and scrimmage-based game play. For matches, accelerometer data were obtained from the beginning of the first quarter to the end of the final quarter, inclusive of stoppages, time-outs and inter-quarter breaks.

Manufacturer software (v6.13.4; Actigraph, FL) was used to download raw accelerometer data, and data were processed using custom code in MATLAB (R2018b; MathWorks, MA). Raw accelerometer data were filtered using a fourth-order band-pass Butterworth filter with cut-off frequencies of 0.1 Hz and 15 Hz (Staunton et al., 2017). AvF_NET_ was used to quantify activity intensity by multiplying resultant acceleration by the player's body mass, and activity volume was calculated as activity intensity multiplied by activity duration (impulse; Staunton et al., 2017; Palmer et al., 2021). These metrics have demonstrated construct validity in basketball when compared to predicted AvF_NET_ calculated from 2-D movement speeds in a basketball exercise simulation test, and were chosen due to their ability to quantify three-dimensional movement and stronger convergent validity against overground running speed than PlayerLoad™ (Staunton et al., 2017).

AvF_NET_ and estimated oxygen uptake ($\dot{V}O_{2}$) for each speed of the Yo-Yo IR1 Test were used to calculate individualized linear relationships between AvF_NET_ and $\dot{V}O_{2}$ (Staunton et al., 2018a). Relative exercise intensity bands based on percentage $\dot{V}O_{2}$ reserve ($\dot{V}O_{2}$R) were then determined for each player to enable quantification of the volume of activity performed at different intensities. Exercise intensity bands were defined as inactive ( ≤ 10% $\dot{V}O_{2}$R), light (>10–40% $\dot{V}O_{2}$R), moderate-vigorous (>40–90% $\dot{V}O_{2}$R), maximal (>90–100% $\dot{V}O_{2}$R), and supramaximal (>100% $\dot{V}O_{2}$R) (Palmer et al., 2021).

Player roles per match were defined as starters (players who started the match on the court), in-rotation bench players (bench players who played 10 or more minutes in a match; Clay and Clay, 2014), and out-rotation bench players (remaining bench players; Palmer et al., 2021). Players' minutes played data were retrieved from league websites (Genius Sports Group, 2019). For training sessions, players were classified as the role they most often played throughout the season. Training and match demands were compared between the regular season and finals (single-elimination format) with consideration for player role and team. Average training session and cumulative weekly training durations were calculated to determine if training schedule differed between the regular season and finals.

### Statistical analyses

Statistical analyses were conducted using SPSS Statistics (v26; IBM Corporation, Armonk, NY) with significance set at *p* ≤ 0.05. Shapiro-Wilk tests indicated several variables were not normally distributed. Data were therefore log-transformed prior to parametric analysis and descriptive data were presented as median (lower quartile—upper quartile). Prior to log-transformation, an offset of 1 was added to variables containing zero values (minutes played, absolute and relative maximal and supramaximal activity) to enable log-transformation of all data points. To assess differences in training and match demands between season periods, and to determine if these differences are dependent on either player role or team, linear mixed models with Bonferroni *post-hoc* tests were conducted with the participant as subjects, and season period (regular season/finals), player role (starter/in-rotation bench/out-rotation bench) and team as the fixed factors. If a significant 3-way interaction effect between season period, player role and team was found, players were separated by player role and team for analyses. When team did not significantly interact with season period in either 3-way or 2-way interaction effects, team was removed as a factor from the model, and the linear mixed model was re-run. When the model was run with only season period and role as fixed factors, significant 2-way interaction effects between season period and player role were followed by linear mixed models for each role separately. If no significant 3-way interaction effect was found, but a significant 2-way interaction between season period and team was found, the interaction effect was reported, however was not followed up with simple main effect analyses due to inter-team comparisons being out of the scope of this investigation. If no significant 3-way or 2-way interaction effects were found, the season period main effect was consulted. Mean differences (MD) and 95% confidence intervals between the regular season and finals were calculated using a bootstrapping technique (randomly 1,000 bootstrap samples; Teixeira et al., 2022). Partial eta squared $\eta_{\text{p}}^{2}$ effect sizes were calculated for the linear mixed model analyses (Lakens, 2013), categorized as follows: 0.01–0.04: small, >0.04–0.14: medium, >0.14: large (Richardson, 2011). Effect sizes (ES) for pairwise comparisons were calculated on log-transformed data using the Effect Sizes and Confidence Intervals module in Jamovi (v2.2.5; Sydney, Australia) and presented as Cohen's d, categorized as follows: <0.2: trivial, 0.2–0.6: small, >0.6–1.2: moderate, >1.2–2.0: large, >2.0: very large (Hopkins, 2002).

## Results

**Figure 1:**
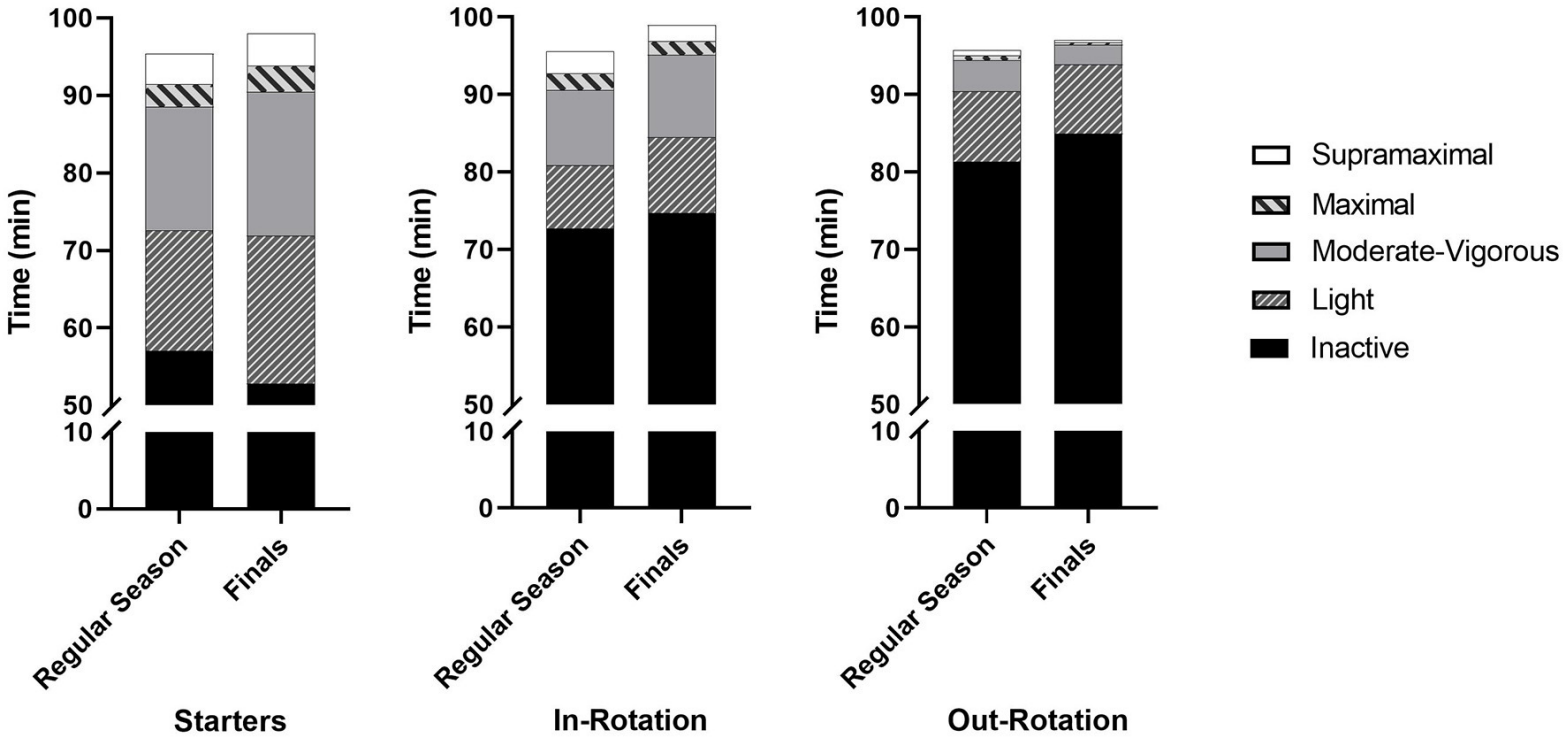
Absolute match time spent in each intensity band during the regular season and finals for each player role.

Match analyses resulted in 387 individual observations during the regular season and 75 observations during finals. Training analyses resulted in 445 individual observations during the regular season and 113 observations during finals. Average match activity for each player role and for all roles together during the regular season and finals are shown in Table 2. Absolute match time spent in each intensity band during the regular season and finals for each player role is presented in Figure 1. Patterns in match activity between the regular season and finals were dependent on both team and player role for the proportion of on-court time spent performing maximal (*F*_(2,342)_ = 3.25; *p* = 0.04, $\eta_{\text{p}}^{2}$ = 0.02) and supramaximal activity (*F*_(2,342)_ = 3.11; *p* = 0.05, $\eta_{\text{p}}^{2}$ = 0.02). In the women's team, bench players out of the main rotation performed less relative maximal [regular season: 0.58% (0.45–0.70), finals: 0.11% (0.06–0.31), MD = −0.32% (−0.50 to −0.16%), *p* = 0.02, ES = 1.23] and supramaximal [regular season: 0.71% (0.55–0.82), finals: 0.10% (0.02–0.41), MD = −0.33% (−0.61 to −0.04%), *p* = 0.04, ES = 0.98] activity in finals compared to the regular season.

**Table 2:** On-court activity during matches in the regular season and finals.

		**Starters**		**In-rotation**		**Out-rotation**		**All roles together**		Team *season period interaction
		**Regular season** **(*n* = 189)**	**Finals** **(*n* = 35)**	**Regular season** **(*n* = 75)**	**Finals** **(*n* = 9)**	**Regular season** **(*n* = 123)**	**Finals** **(*n* = 31)**	**Regular season** **(*n* = 387)**	**Finals** **(*n* = 75)**	
Playing time	min	29.2 (26.4–35.3)	32.6* (31.5–38.1)	16.3 (15.9–22.5)	20.1 (12.1–22.0)	4.1 (3.1–5.6)	1.1* (0.1–5.5)	16.2 (4.4–28.4)	16.7 (1.6–32.4)	NS
AvF_NET_	N	343 (283–365)	360 (307–410)	223 (171–283)	220 (205–250)	137 (99–160)	129 (83–161)	215 (145–306)	213 (134–317)	NS
Impulse	kN·s	1,939 (1,499–2,149)	2,115 (1,574–2,474)	1,386 (973–1,580)	1,201 (1,171–1,456)	778 (530–979)	764 (448–972)	1,218 (782–1,737)	1,201 (780–1,645)	NS
Inactive	min	57.0 (40.1-63.1)	52.8 (36.9-61.5)	72.7 (67.6-80.6)	74.7 (61.8-81.9)	81.3 (74.8-86.6)	84.9 (74.5-91.1)	75.2 (64.0-83.0)	74.9 (60.0-86.8)	*p* < 0.01
	%	60.5 (41.5–63.9)	55.7* (37.3–59.3)	75.8 (69.1–79.7)	74.0 (67.2–79.3)	85.9 (80.2–88.9)	88.6 (82.4–91.7)	79.4 (65.3–86.1)	79.3 (62.0–89.1)	*p* < 0.01
Light	min	15.6 (12.1–30.7)	19.1 (13.9–34.0)	8.1 (6.8–15.8)	9.8 (7.4–15.4)	9.1 (5.7–11.8)	9.0 (5.0–11.5)	10.2 (7.3–14.8)	10.3 (6.6–17.1)	NS
	%	15.7 (12.7–34.0)	18.5 (14.1–37.3)	8.5 (6.7–16.0)	10.5 (7.2–15.3)	9.5 (6.4–12.3)	9.3 (5.8–11.2)	10.2 (7.6–15.3)	10.7 (6.4–17.4)	NS
Moderate-vigorous	min	15.9 (14.6–16.8)	18.6 (15.8–19.8)	9.7 (8.9–10.6)	10.6 (7.4–12.5)	4.0 (3.3–4.7)	2.5 (1.6–4.5)	5.5 (3.7–13.5)	5.7 (2.3–13.9)	NS
	%	16.1 (14.9–19.3)	18.0 (15.4–22.8)	9.8 (9.0–11.0)	10.8 (7.2–13.2)	4.2 (3.2–4.8)	2.9 (1.7–4.4)	5.6 (4.1–13.6)	5.5 (2.6–14.1)	NS
Maximal	min	2.9 (2.0–3.2)	3.4* (2.2–3.9)	2.1 (1.2–2.7)	1.7 (1.6–3.2)	0.6 (0.5–0.8)	0.3* (0.1–0.8)	0.9 (0.6–2.4)	1.0 (0.1–2.7)	NS
	%	3.2 (2.1–3.5)	3.6 (2.2–4.0)	2.1 (1.2–2.8)	1.7 (1.6–3.5)	0.6 (0.5–0.8)	0.3 (0.1–0.8)	0.9 (0.6–2.5)	1.0 (0.1–2.7)	NS
Supramaximal	min	3.9 (3.2–4.3)	4.2 (3.6–4.6)	2.8 (1.6–3.6)	2.1 (1.9–4.2)	0.7 (0.6–1.3)	0.3* (0.0–1.0)	1.5 (0.7–3.6)	1.3 (0.2–4.0)	NS
	%	4.4 (3.2–4.5)	4.4 (3.5–5.0)	2.8 (1.7–3.7)	2.0 (1.9–4.6)	0.7 (0.7–1.3)	0.3 (0.0–1.1)	1.5 (0.7–3.7)	1.3 (0.2–3.9)	NS

Values are presented as median (lower quartile—upper quartile).

NS represents not significant, * represents a significant difference to the regular season (p < 0.05), min represents absolute time in minutes, % represents proportion of session time.

Patterns in match activity between the regular season and finals were not dependent on both team and player role for the remaining variables (*F*_(2,342−462)_ = 0.13–2.85; *p* = 0.06–0.88, $\eta_{\text{p}}^{2}$ < 0.01–0.02). Player role influenced patterns of activity between the regular season and finals for minutes played (*F*_(2,462)_ = 7.74; *p* < 0.01, $\eta_{\text{p}}^{2}$ = 0.05), absolute maximal activity (*F*_(2,342)_ = 5.68; *p* < 0.01, $\eta_{\text{p}}^{2}$ = 0.04), absolute supramaximal activity (*F*_(2,342)_ = 3.45; *p* = 0.03, $\eta_{\text{p}}^{2}$ = 0.03) and relative time spent being inactive (*F*_(2,342)_ = 5.22; *p* < 0.01, $\eta_{\text{p}}^{2}$ = 0.03). For starters, finals resulted in more minutes played [MD = 4.4 min (2.5–6.3 min); *p* < 0.01; ES = 0.83], more absolute maximal activity [MD = 0.4 min (0.2–0.7 min); *p* = 0.04; ES = 0.34] and less relative time spent being inactive [MD = –4.9% (−7.8 to −2.1%), *p* = 0.02, ES = 0.38] when compared to the regular season. For bench players out of the main rotation, finals resulted in fewer minutes played [MD = −2.1 min (−3.3 to –0.8 min), *p* = 0.01, ES = 1.09], less absolute maximal activity [MD = −0.2 min (−0.4 to −0.1 min), *p* = 0.02, ES = 0.83] and less absolute supramaximal activity [MD = −0.4 min (−0.6 to −0.2 min), *p* = 0.05, ES = 1.09] when compared to the regular season.

Despite significant 2-way interaction effects between season period and player role for absolute time spent being inactive, no differences between the regular season and finals were found when assessing player roles separately (*p* = 0.06–0.30). Differences in absolute (*F*_(2,342)_ = 7.38; *p* < 0.01, $\eta_{\text{p}}^{2}$ = 0.02) and relative (*F*_(2,342)_ = 4.29; *p* = 0.04, $\eta_{\text{p}}^{2}$ = 0.01) time spent being inactive between the regular season and finals were dependent on team.

**Figure 2:**
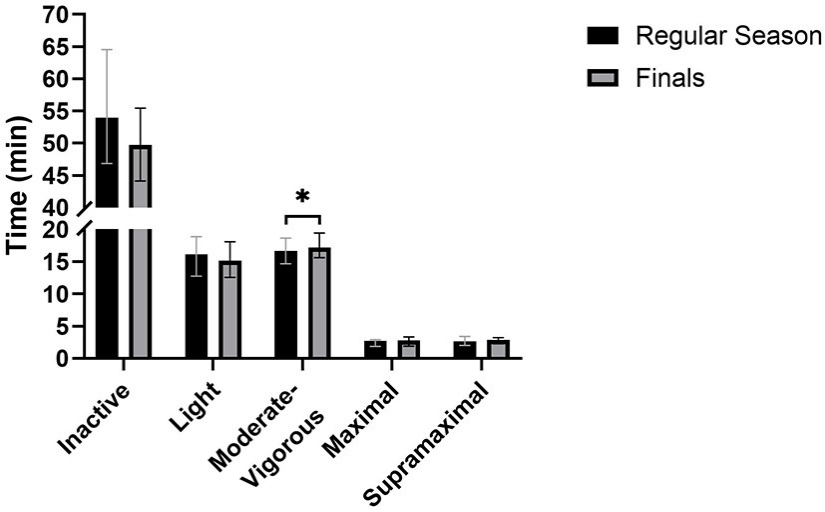
Absolute training time spent in each intensity band during the regular season and finals. *Represents a significant difference between the regular season and finals. Error bars represent the upper and lower quartiles.

Average training session durations [regular season: 90 min (86–100), finals: 84 min (81–91)], and average cumulative weekly training durations [regular season: 172 min (111–184), finals: 166 min (118–172)] were not different between the regular season and finals. Average on-court training activity for all roles together during the regular season and finals are shown in Table 3. Absolute training time spent in each intensity band during the regular season and finals is presented in Figure 2. Differences in training activity between the regular season and finals were not dependent on team and player role (season period x player role x team interaction effect: *F*_(1,475−558)_ = 0.02–1.2; *p* = 0.27–0.88, $\eta_{\text{p}}^{2}$ < 0.01). Differences in absolute time spent being inactive (*F*_(1,475−558)_ = 4.94; *p* = 0.03, $\eta_{\text{p}}^{2}$ = 0.01) and relative time spent performing moderate-vigorous (*F*_(1,475−558)_ = 5.55; *p* = 0.02, $\eta_{\text{p}}^{2}$ = 0.01) and supramaximal (*F*_(1,475−558)_ = 4.10; *p* = 0.04, $\eta_{\text{p}}^{2}$ = 0.01) activity between the regular season and finals were dependent on team. Differences in training activity between the regular season and finals for the remaining variables were not dependent on player role (season period x player role interaction effect: *F*_(2,475–558)_ = 0.06–1.01; *p* = 0.37–0.94, $\eta_{\text{p}}^{2}$ < 0.01) or team (*F*_(2,475−558)_ < 0.01–3.49; *p* = 0.06–0.95, $\eta_{\text{p}}^{2}$ ≤ 0.01). Average training intensity [MD = 15 N (0 to 30 N), *p* = 0.02; ES = 0.20], absolute time spent performing moderate-vigorous activity [MD = 0.5 min (−0.2 to 1.1 min), *p* = 0.04; ES = 0.16] and relative time spent performing maximal activity [MD = 0.3% (0.1 to 0.6%), *p* = 0.01; ES = 0.14) were greater in finals compared to the regular season, while relative time spent being inactive was lower in finals compared to the regular season [MD = −2.7% (−5.0 to −0.4%); *p* = 0.03; ES = 0.25].

**Table 3:** On-court activity during training sessions in the regular season and finals.

		**Regular season (*n* = 445)**	**Finals (*n* = 113)**	Team *Season period interaction effect
AvF_NET_	N	315 (284–371)	321* (272–395)	NS
Impulse	kN·s	1,803 (1,463–2,215)	1,812 (1,456–2,260)	NS
Inactive	min	54.0 (46.9–64.6)	49.7 (44.1–55.4)	*p* = 0.03
	%	59.8 (52.6–63.1)	56.3* (46.9–60.2)	NS
Light	min	16.1 (12.8–18.9)	15.2 (12.5–18.1)	NS
	%	16.4 (13.5–20.0)	16.6 (13.1–21.1)	NS
Moderate-vigorous	min	16.6 (14.7–18.7)	17.2* (15.6–19.5)	NS
	%	16.9 (15.4–20.3)	19.3 (18.1–22.3)	*p* = 0.02
Maximal	min	2.7 (1.9–2.9)	2.8 (1.9–3.3)	NS
	%	2.6 (2.1–3.5)	3.3* (2.2–3.7)	NS
Supramaximal	min	2.7 (2.0–3.4)	2.9 (2.3–3.2)	NS
	%	2.8 (2.1–3.3)	3.1 (2.5–3.9)	*p* = 0.04

Values are presented as median (lower quartile—upper quartile).

NS represents not significant, * represents a significant difference to the regular season (p < 0.05), min represents absolute time in minutes, % represents proportion of session time.

## Discussion

Starters played more minutes and subsequently performed more maximal activity and spent less time being inactive during finals matches when compared to the regular season. Bench players out of the main rotation played fewer minutes and performed less maximal and supramaximal activity in finals compared to the regular season. Training sessions in finals elicited a greater average intensity, more absolute moderate-vigorous activity, more relative maximal activity, and less relative inactive time compared to the regular season. These findings supported the hypothesis that match demands for starters increased in finals compared to the regular season. The hypothesis that match demands for bench players was less in finals than the regular season was supported by the out-rotation bench group, but not by the in-rotation bench group. The hypothesis that training demands would be greater in finals for starters was partially supported, however this finding was not dependent on player role as anticipated.

During finals matches, starters played 4.4 more minutes than the regular season. Subsequently, starters performed 14% more absolute maximal activity, and spent 8% less relative time being inactive in finals than the regular season. This finding likely reflects the coach wanting to keep their best players on the court in finals to increase the team's chances of winning with greater match difficulty and importance (Clay and Clay, 2014). It is therefore suggested that starters need to be physically prepared for increased match demands during finals, and might need to perform additional maximal-intensity activity throughout the regular season. Starters might also require additional recovery during finals to limit fatigue carried into subsequent matches. However, the finding that supramaximal activity was not different between finals and regular season might suggest that the starters in this study were not conditioned enough to perform more supramaximal activity than usual, and that the extra time on-court was spent performing maximal activity instead. Greater maximal activity in finals when compared to regular season aligns with other seasonal team sports (e.g., Australian and Gaelic football), where distance covered and high-intensity activity increased in finals compared to the regular season (Aughey, 2011; Mangan et al., 2019). However, these findings do not align with previous research in professional basketball, where match sRPE was not different between the regular season and finals (Ferioli et al., 2021). It is possible the aforementioned study did not identify differences between the regular season and finals because all players (*n* = 10) were analyzed together, without considering player role. This notional explanation is supported by the present study findings, which showed that differences in multiple variables between the regular season and finals were dependent on player role. Specifically, in finals compared to the regular season, playing time and match demands for starters was higher, while playing time and match demands for bench players out of the main rotation was lower. It is therefore possible that when all player roles are combined, the starters' increase in demands offsets the decrease in demands for out-rotation bench players, resulting in few differences between the regular season and finals. This comparison highlights the importance of considering player role when quantifying match demands in basketball.

In contrast to starters, bench players out of the main rotation played 2.1 fewer minutes in finals than the regular season, and subsequently performed 33–57% less absolute maximal and supramaximal activity in finals. This is again likely due to the coach allocating more playing time to the best players, resulting in the bottom players receiving less playing time. For athletes competing in seasonal sport, it has been suggested that players who train once or twice a week and play minimal minutes in matches might not receive sufficient stimuli to maintain adequate conditioning in-season (Joyce and Lewindon, 2014). Additionally, starting players have been reported to possess superior aerobic conditioning when compared to bench players (Scanlan et al., 2021), which is potentially caused or amplified by the extra in-game conditioning starting players receive compared to bench players (Palmer et al., 2021; Russell et al., 2021). Therefore, bench players out of the main rotation who receive reduced playing time during a finals series might need to supplement their conditioning training during finals with extra maximal and supramaximal-intensity activity to ensure their conditioning levels are not further reduced compared to starters (Joyce and Lewindon, 2014). Maintenance of conditioning is important in case an injury to a regular starter or in-rotation bench player, or change in strategy, requires an out-rotation bench player to play more minutes. Results in the present study showed that training demands were not adapted throughout the season to meet the role demands of players.

During training sessions, the average training intensity, absolute moderate-vigorous activity and relative maximal activity were 3–12% higher, while relative time spent being inactive was 5% lower in finals when compared to the regular season. During the regular season, coaches might have spent more training time teaching content, where players are inactive listening to instructions, whereas in finals, teams might have spent more training time practicing the content they already know, resulting in more active time. The increased training intensity might have also been created by contextual factors, such as players being more motivated to train harder in finals knowing the consequences of wins and losses are greater, and knowing that playing time might be more difficult to earn (Kempton et al., 2015). Previous researchers have demonstrated a greater sRPE during training sessions greater than 24 h from matches in the regular season compared to finals (Ferioli et al., 2021). It is possible that the differences in findings between studies reflect differences in metrics used, or differences in competition level and finals series format. In the present study, teams were semi-professional and therefore typically played matches on weekends, with occasional matches on Thursdays and Fridays. This match schedule was consistent between the regular season and finals. In the previous study on professional basketball, regular season matches were played once a week, whereas in finals, matches were played approximately every 2–3 days (Ferioli et al., 2021). It might be expected that due to the reduced time for recovery between matches in finals, training intensity and volume would be intentionally reduced, whereas in the present study, the match schedule stayed relatively consistent. As tapering training volume prior to important competitions is recommended (Gamble, 2006), when the match schedule is consistent between the regular season and finals coaches might need to consider shortening training durations in finals given the tendency for training intensity to increase, as demonstrated by the present study.

While this study investigated aspects of semi-professional training and match demands not previously explored in basketball research, it is acknowledged that these results might not be representative of other teams or competitions. While it is possible that the findings were only representative of the two teams used in the study, as most of the main findings were not dependent on team, they are more generalizable than other studies in elite sport research where only one team was used (Aughey, 2011; Ferioli et al., 2021), especially given that both sexes were included. To confirm this generalizability, further research could be conducted on a larger sample across multiple competitions. Further research could also focus on quantifying intra-seasonal changes in training and match demands in other league formats, such as the National Basketball Association (NBA) where the season is substantially longer, matches are more frequent, and teams regularly travel interstate (Esteves et al., 2021). Additionally, the pattern of changes in training and match demands throughout a season might differ depending on factors such as tactical considerations and coach strategy, fixture scheduling, player fitness, player motivation, and the coach's understanding of training periodization recommendations (Robertson and Joyce, 2015). Future research could use an interdisciplinary approach to investigate the potential contextual factors (Pino-Ortega et al., 2019) and specific causes of changes in training and match demands throughout a season. Lastly, while this study investigated differences in accelerometry-based time-motion analysis demands between the regular season and finals, future research could assess differences in behavioral and spatial variables and technical and tactical factors (Arede et al., 2021).

## Conclusion

This study showed that in finals matches in semi-professional basketball, starters received more playing time and performed more maximal activity while spending less time being inactive compared to the regular season. Bench players out of the main rotation received less playing time and performed less maximal and supramaximal activity than the regular season. However, during training sessions a team-wide prescription of physical demands occurs, where more time was spent performing moderate-vigorous and maximal activity and less time was spent being inactive in finals compared to the regular season. These findings suggest that in semi-professional basketball, starters need to be physically prepared for an increase in match demands in finals. Additionally, bench players out of the main rotation might need to supplement their training in finals with extra maximal and supramaximal activity to maintain their conditioning levels. Coaches and physical preparation staff can use these findings to prepare their players for training sessions and matches during finals to maintain optimal performance and minimize injury risk, with specific application to semi-professional basketball.

## Data availability statement

The raw data supporting the conclusions of this article will be made available by the authors, without undue reservation.

## Ethics statement

This study involving human participants was reviewed and approved by La Trobe University Human Research Ethics Committee. Written informed consent to participate in this study was provided by the participants or the participants' legal guardian/next of kin, where participants were less than 18 years of age.

## Author contributions

Conceptualization, methodology, resources, writing—review and editing, project administration, and visualization: JP, DW, RB, and MK. Software: JP and MK. Validation, formal analysis, and investigation: JP with assistance from DW, RB, and MK. Data curation and writing—original draft preparation: JP. Supervision: DW, RB, and MK. Funding acquisition: MK. All authors have read and agreed to the published version of the manuscript.

## Conflict of interest

The authors declare that the research was conducted in the absence of any commercial or financial relationships that could be construed as a potential conflict of interest.

## Publisher's note

All claims expressed in this article are solely those of the authors and do not necessarily represent those of their affiliated organizations, or those of the publisher, the editors and the reviewers. Any product that may be evaluated in this article, or claim that may be made by its manufacturer, is not guaranteed or endorsed by the publisher.

## References

[B1] Abdelkrim N. B. Castagna C. Jabri I. Battikh T. El Fazaa S. El Ati J. (2010). Activity profile and physiological requirements of junior elite basketball players in relation to aerobic-anaerobic fitness. J. Strength Cond. Res. 24, 2330–2342. 10.1519/JSC.0b013e3181e381c1 20802281

[B2] Arede J. Cumming S. Johnson D. Leite N. (2021). The effects of maturity matched and un-matched opposition on physical performance and spatial exploration behavior during youth basketball matches. PLoS ONE 16, e0249739. 10.1371/journal.pone.0249739 33831106 PMC8031392

[B3] Aughey R. J. (2011). Increased high-intensity activity in elite Australian football finals matches. Int. J. Sports Physiol. Perform. 6, 367–379. 10.1123/ijspp.6.3.367 21911862

[B4] Clay C. D. Clay E. K. (2014). Player rotation, on-court performance and game outcomes in NCAA men's basketball. Int. J. Perform. Anal. Sport 14, 606–619. 10.1080/24748668.2014.11868746

[B5] Esteves P. T. Mikolajec K. Schelling X. Sampaio J. (2021). Basketball performance is affected by the schedule congestion: NBA back-to-backs under the microscope. Eur. J. Sport Sci. 21, 26–35. 10.1080/17461391.2020.1736179 32172667

[B6] Ferioli D. Scanlan A. T. Conte D. Tibiletti E. Rampinini E. (2021). The business end of the season: a comparison between playoff and regular-season workloads in professional basketball players. Int. J. Sports Physiol. Perform. 16, 655–662. 10.1123/ijspp.2020-0405 33561821

[B7] Ferioli D. Schelling X. Bosio A. La Torre A. Rucco D. Rampinini E. (2020). Match activities in basketball games: comparison between different competitive levels. J. Strength Cond. Res. 34, 172–182. 10.1519/JSC.0000000000003039 30741861

[B8] Gabbett T. J. Hulin B. T. Blanch P. Whiteley R. (2016). High training workloads alone do not cause sports injuries: how you get there is the real issue. Br. J. Sports Med. 50, 444. 10.1136/bjsports-2015-095567 26795610

[B9] Gamble P. (2006). Periodization of training for team sports athletes. Strength Cond. J. 28, 56. 10.1519/00126548-200610000-00009

[B10] Genius Sports Group (2019). NBL1. Available online at: http://fibalivestats.com (accessed June 18, 2020).

[B11] Hopkins W. (2002). A Scale of Magnitudes for Effect Statistics. A New View of Statistics. Available online at: http://sportsci.org/resource/stats/effectmag.html (accessed July 28, 2018).

[B12] Joyce D. Lewindon D. (2014). High-Performance Training for Sports. Human Kinetics, Incorporated.

[B13] Kelly S. J. Murphy A. J. Watsford M. L. Austin D. Rennie M. (2015). Reliability and validity of sports accelerometers during static and dynamic testing. Int. J. Sports Physiol. Perform. 10, 106–111. 10.1123/ijspp.2013-0408 24911138

[B14] Kempton T. Sullivan C. Bilsborough J. C. Cordy J. Coutts A. J. (2015). Match-to-match variation in physical activity and technical skill measures in professional Australian Football. J. Sci. Med. Sport 18, 109–113. 10.1016/j.jsams.2013.12.006 24444753

[B15] Lakens D. (2013). Calculating and reporting effect sizes to facilitate cumulative science: a practical primer for t-tests and ANOVAs. Front. Psychol. 4, 863. 10.3389/fpsyg.2013.00863 24324449 PMC3840331

[B16] Mangan S. Ryan M. Shovlin A. Mcgahan J. Malone S. O'neill C.. (2019). Seasonal changes in Gaelic football match-play running performance. J. Strength Cond. Res. 33, 1685–1691. 10.1519/JSC.0000000000002269 31125327

[B17] Nicolella D. P. Torres-Ronda L. Saylor K. J. Schelling X. (2018). Validity and reliability of an accelerometer-based player tracking device. PLoS ONE 13, e0191823. 10.1371/journal.pone.0191823 29420555 PMC5805236

[B18] Palmer J. Wundersitz D. Bini R. Kingsley M. (2021). Effect of player role and competition level on player demands in basketball. Sports 9, 38. 10.3390/sports9030038 33800459 PMC8002055

[B19] Petway A. J. Freitas T. T. Calleja-Gonzalez J. Medina Leal D. Alcaraz P. E. (2020). Training load and match-play demands in basketball based on competition level: a systematic review. PLoS ONE 15, e0229212. 10.1371/journal.pone.0229212 32134965 PMC7058381

[B20] Pino-Ortega J. Rojas-Valverde D. Gómez-Carmona C. D. Bastida-Castillo A. Hernández-Belmonte A. García-Rubio J.. (2019). Impact of contextual factors on external load during a congested-fixture tournament in elite U'18 basketball players. Front. Psychol. 10, 1100. 10.3389/fpsyg.2019.01100 31156514 PMC6529817

[B21] R Core Team (2021). The Comprehensive R Archive Network. R Core Team, Vienna, Austria. Available online at: https://cran.r-project.org/

[B22] RDocumentation (2022). Samplesize_Mixed: Sample Size for Linear Mixed Models. Available online at: https://www.rdocumentation.org/packages/sjstats/versions/0.18.1/topics/samplesize_mixed (accessed July 27, 2022).

[B23] Richardson J. T. (2011). Eta squared and partial eta squared as measures of effect size in educational research. Educ. Res. Rev. 6, 135–147. 10.1016/j.edurev.2010.12.001

[B24] Robertson S. J. Joyce D. G. (2015). Informing in-season tactical periodisation in team sport: development of a match difficulty index for Super Rugby. J. Sports Sci. 33, 99–107. 10.1080/02640414.2014.925572 24977714

[B25] Russell J. L. Mclean B. D. Stolp S. Strack D. Coutts A. J. (2021). Quantifying training and game demands of a National Basketball Association Season. Front. Psychol. 12, 5782. 10.3389/fpsyg.2021.793216 34992569 PMC8724530

[B26] Scanlan A. Dascombe B. Reaburn P. (2011). A comparison of the activity demands of elite and sub-elite Australian men's basketball competition. J. Sports Sci. 29, 1153–1160. 10.1080/02640414.2011.582509 21777151

[B27] Scanlan A. T. Stojanović E. Milanović Z. Teramoto M. Jeliči,ć M. Dalbo V. J. (2021). Aerobic capacity according to playing role and position in elite female basketball players using laboratory and field tests. Int. J. Sports Physiol. Perform. 16, 435–438. 10.1123/ijspp.2019-1001 33406486

[B28] Staunton C. Wundersitz D. Gordon B. Custovic E. Stanger J. Kingsley M. (2018b). The effect of match schedule on accelerometry-derived exercise dose during training sessions throughout a competitive basketball season. Sports 6, 69. 10.3390/sports6030069 30041486 PMC6162803

[B29] Staunton C. Wundersitz D. Gordon B. Kingsley M. (2017). Construct validity of accelerometry-derived force to quantify basketball movement patterns. Int. J. Sports Med. 38, 1090–1096. 10.1055/s-0043-119224 28965347

[B30] Staunton C. Wundersitz D. Gordon B. Kingsley M. (2018a). Accelerometry-derived relative exercise intensities in elite women's basketball. Int. J. Sports Med. 39, 822–827. 10.1055/a-0637-9484 29986346

[B31] Stojanović E. Stojiljković N. Scanlan A. T. Dalbo V. J. Berkelmans D. M. Milanović Z. (2018). The activity demands and physiological responses encountered during basketball match-play: a systematic review. Sports Med. 48, 111–135. 10.1007/s40279-017-0794-z 29039018

[B32] Teixeira J. E. Alves A. R. Ferraz R. Forte P. Leal M. Ribeiro J.. (2022). Effects of chronological age, relative age, and maturation status on accumulated training load and perceived exertion in young sub-elite football players. Front. Physiol. 547. 10.3389/fphys.2022.832202 35432006 PMC9010324

[B33] Wundersitz D. W. Josman C. Gupta R. Netto K. J. Gastin P. B. Robertson S. (2015). Classification of team sport activities using a single wearable tracking device. J. Biomech. 48, 3975–3981. 10.1016/j.jbiomech.2015.09.015 26472301

[B34] Ziv G. Lidor R. (2009). Physical attributes, physiological characteristics, on-court performances and nutritional strategies of female and male basketball players. Sports Med. 39, 547–568. 10.2165/00007256-200939070-00003 19530751

